# A Fourier-Based Image Formation Algorithm for Geo-Stationary GNSS-Based Bistatic Forward-Looking Synthetic Aperture Radar

**DOI:** 10.3390/s19091965

**Published:** 2019-04-26

**Authors:** Zhangfan Zeng, Zhiming Shi, Sainan Xing, Yongcai Pan

**Affiliations:** School of Computer Science and Information Engineering, Hubei University, Wuhan 430062, China; zengzhangfan@hotmail.com (Z.Z.); shichiminh@outlook.com (Z.S.); panycf@163.com (Y.P.)

**Keywords:** GNSS, forward looking SAR, image formation

## Abstract

A Geo-Stationary GNSS-based Bistatic Forward-Looking Synthetic Aperture Radar (GeoSta-GNSS-BFLSAR) system is a particular kind of passive bistatic SAR system. In this system, a geo-stationary GNSS is used as the transmitter, while the receiver is deployed on a moving aircraft, which travels towards a target in a straight line. It is expected that such a radar system has potential for self-landing, self-navigation and battlefield information acquisition applications, etc. Up to now, little information from a research perspective can be found about GeoSta-GNSS-BFLSAR systems. To address this information gap, this paper proposes a preliminary image formation algorithm for GeoSta-GNSS-BFLSAR. The full details of the mathematical derivation are given. It is highlighted that, to overcome the long dwell time and spatial variance of GeoSta-GNSS-BFLSAR, a modified migration correction factor must be designed. In addition, the system performances and technical limitations of GeoSta-GNSS-BFLSAR such as focusing depth and spatial resolution are analytically discussed. In the end, a set of simulations including the image formation algorithm, focusing depth and spatial resolution were conducted for verification. It is demonstrated that the focusing performances of the proposed algorithm have a high level of similarity with the theoretical counterparts. This article thus proves the feasibility of GeoSta-GNSS-BFLSAR systems from a simulation level and establishes a foundation for the real applications of such a radar scheme in the future.

## 1. Introduction

**Figure 1:**
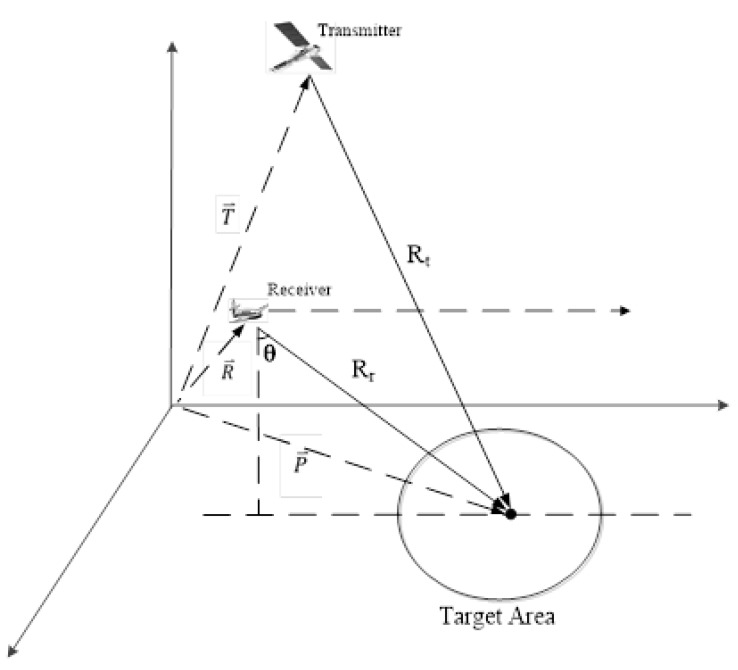
Schematic of GeoSta-GNSS-BFLSAR.

The Geo-Stationary GNSS-based bistatic forward looking SAR (GeoSta-GNSS-BFLSAR) is a bistatic SAR system subclass [1]. It employs geo-stationary GNSS as the illuminator, while the receiver is mounted on a moving aircraft. The aircraft is moving along a straight line and the radar antenna is always looking forward. Such a configuration is expected to have great potential in both civil and military applications fields. With regards to civil applications, GeoSta-GNSS-BFLSAR can be used in remote sensing, Earth observation, ground deformation detection, terrain mapping and forest monitoring, etc. On the other hand, GeoSta-GNSS-BFLSAR can be used in self-landing, self-navigation and battlefield monitoring applications too. In addition, compared with conventional bistatic SAR systems, such a system benefits from good safety, low cost, good system stability, large constellation, short revisit time and a flexible configuration, etc. The schematic of a GeoSta-GNSS-BFLSAR system is shown in Figure 1.

The key problems in GeoSta-GNSS-BFLSAR systems are signal synchronization and image formation. Investigations on signal synchronization have developed over the last decades and a large number of works on the topic can be found [2,3,4,5,6,7,8]. On the other hand, research achievements on GNSS- based SAR imaging formation have been presented in [9,10,11,12,13,14,15,16,17,18,19] from theoretical, experimental and application perspectives. Antoniou et al. first proposed a modified Range-Doppler algorithm for a Galileo-based BSAR system [9]. Hu et al. presented his work on a frequency domain focusing method in Geo-SAR [10]. However, none of them are applicable for a forward-looking scenario. As for high orbit Geo-SAR signal processing, Wang et al. proposed a new radar configuration, where the transmitter is a GEO satellite and the receiver is a LEO satellite. The imaging capability was demonstrated by a modified time domain BPA algorithm [16]. Sun et al. presented the concept and advantages of Geo-BiSAR for the first time, where the spatial resolution, radar signal to noise, etc. were studied [17]. Lazarov et al. proved the feasibility of inverse SAR imaging by using GPS and a fixed receiver [18,19]. It could be observed that, however, these high orbit radar schemes are quite different from GeoSta-GNSS-BFLSAR, both in geometry or peculiarity. Therefore, those image formation algorithms can’t be adopted directly to our radar scheme. As for forward-looking SAR image formation, Qiu et al. proposed a bistatic forward-looking configuration and demonstrated its imaging capability [20]. Experimental demonstration of spaceborne-airborne forward-looking image formation was reported by Espeter et al. in [21], which verified the feasibility of such a configuration. An improved Omega-K algorithm for forward-looking spotlight SAR mode was demonstrated on a simulation basis in [22]. Wu et al. presented a keystone transform-based NLCS image formation algorithm for a bistatic forward-looking geometry in [23]. It is noted that, however, these examples are particularly for low orbit chirp pulse sensors, and hence not suitable for our configuration, where BPSK modulation and a pseudo-random sequence-based continuous wave are employed.

From the discussion mentioned above, it can be seen that little information can be found about GeoSta-GNSS-BFLSAR systems from a research perspective. To address this literature gap, this paper proposes a preliminary image formation algorithm for GeoSta-GNSS-BFLSAR. The new algorithm is implemented in 2-dimensional frequency domain by applying bulk range migration correction (RCM) and azimuth signal compression. It is highlighted that the specially designed migration correction factor is particularly suitable for the long dwell time and spatial variance seen in GeoSta-GNSS-BFLSAR. In addition, analytical derivations in terms of spatial resolution and focusing depth are presented. The proposed algorithm is tested and its simulation and relevant performances are verified as well. It is noted that like any GNSS-based passive bistatic system, synchronization (i.e., spatial synchronization, phase synchronization, time synchronization, etc.), low signal to noise ratio, an ionosphere effects may have a great impact on the imaging performance [24,25,26,27,28], however, they are beyond the scope of this paper and are not discussed here. More details about signal synchronization solutions can be found in [4,5,18,19].

The rest of this paper is organized as follows: the signal model and radar performance theories are presented in Section 2. The proposed imaging algorithm is introduced in Section 3. Section 4 provides the simulation results and performance verification. The conclusions are given in Section 5.

## 2. GeoSta-GNSS-BFLSAR Signal Model and Analysis

### 2.1. GeoSta-GNSS-BFLSAR Echo SignalModel

In GeoSta-GNSS-BFLSAR systems, the transmitted ranging signal is used as the radar signal. Taking the GPS L1 band signal as an example, it is a BPSK modulated continuous wave. Soon after being reflected from the target, the signal is received by the GeoSta-GNSS-BFLSAR ground receiver. After demodulation, the received signal is given by [1]:

(1) $$s\left(t,\eta \right)=\sigma \cdot p\left[t-R\left(\eta \right)/c\right]\cdot exp\left\{-j2\pi f_{c}\left[t-R\left(\eta \right)\right]/c\right\}$$

where λ is the carrier wavelength, t and η are the fast time and slow time, respectively. p[·] is the envelope of the received signal. σ is the back-scattering reflection coefficient. R(·) is the round-trip distance of a point target, which is expressed as:

(2) $$R\left(\eta \right)=R_{t}+R_{r}\left(\eta \right)$$

where $R_{t}$ is the distance between the transmitter and the target, while $R_{r}\left(\eta \right)$ is that between the receiver and the target, respectively given by:

$$R_{t}=\left|\vec{T}-\vec{P}\right|$$

(3) $$R_{r}\left(\eta \right)=\sqrt{R_{c}{}^{2}cos\theta^{2}+v^{2}{\left(\eta -\frac{R_{c}sin\theta }{v}\right)}^{2}}$$

where $\vec{T}$ and $\vec{P}$ are the vectors of the satellite position and point target, $\;R_{c}$ is the distance between the receiver and target at the starting point, v is the moving velocity of the receiver platform, and θ is the inclination angle at the starting point.

### 2.2. GeoSta-GNSS-BFLSAR 2 Dimensional Spectrum

In this section, the 2-dimensional spectrum of a GeoSta-GNSS-BFLSAR received signal is derived on the basis of the echo signal, i.e., Equation (1), by introducing the Point of Stationary Phase (POSP) method. Applying a Taylor Expansion, the slant range between receiver and target, i.e., $R_{r}\left(\eta \right),$ could be approximated as [1]:

(4) $$R_{r}\left(\eta \right)=\sqrt{R_{c}{}^{2}+v^{2}\eta^{2}-2R_{c}v\eta sin\theta }≃R_{c}cos\left[1+\frac{v^{2}{\left(\eta -\frac{R_{c}sin\theta }{v}\right)}^{2}}{2R_{c}{}^{2}cos\theta^{2}}\right]$$

As such, substituting Equation (4) into Equation (1), the received signal is expressed as:

(5) $$s\left(t,\eta \right)=\sigma \cdot p\left[t-R\left(\eta \right)/c\right]\cdot exp\left\{-j2\pi f_{c}\left\{R_{c}cos\theta \left[1+\frac{v^{2}{\left(\eta -\frac{R_{c}sin\theta }{v}\right)}^{2}}{2R_{c}{}^{2}cos\theta^{2}}\right]\right\}+\left|\vec{T}-\vec{P}\right|/c\right\}$$

The 2-dimensional spectrum of the received signal can be obtained by applying a Fourier transformation on Equation (5) in the range direction and azimuth direction one after another. Firstly, the Fourier transformation is performed on Equation (5) in the range direction, and the resulting output is given as:

(6) $$S\left(f_{t},\eta \right)=\sigma \cdot P\left(f_{t}\right)\cdot exp\left[-j2\pi \left(f_{c}+f_{t}\right)\frac{R\left(\eta \right)}{c}\right]$$

Secondly, after applying a Fourier transformation on Equation (6) in the azimuth direction, the 2-dimensional spectrum GeoSta-GN-SS-BFLSAR can be expressed as:

$$S\left(f_{t},f_{\eta }\right)=\sigma \cdot P\left(f_{t},f_{\eta }\right)\cdot exp\left\{2\pi j\frac{f_{c}\left(R_{c}cos\theta +\left|\vec{T}-\vec{P}\right|\right)}{c}\right\}$$

$$exp\left\{2\pi j\frac{cR_{c}cos\theta }{2v^{2}\left(f_{c}+f_{t}\right)}f_{\eta }{}^{2}-2\pi j\frac{f_{t}\left(R_{c}cos\theta +\left|\vec{T}-\vec{P}\right|\right)}{c}\right\}$$

(7) $$exp\left(-2\pi jf_{\eta }\frac{R_{c}}{v}sin\theta \right)$$

From Equation (7), it could be observed that, the 2-dimensional spectrum of the echo signal of GeoSta-GNSS-BFLSAR is constituted by three terms. The first term $\frac{f_{c}\left(R_{c}cos\theta +\left|\vec{T}-\vec{P}\right|\right)}{c}$ is the constant phase, which only includes the effective radar information of the echo signal. The second term $\frac{cR_{c}cos\theta }{2v^{2}\left(f_{c}+f_{t}\right)}f_{\eta }{}^{2}-\frac{f_{t}\left(R_{c}cos\theta +\left|\vec{T}-\vec{P}\right|\right)}{c}$ is the sum of the transmitter’s range migration and the receiver’s range shift. The last term $f_{\eta }\frac{R_{c}}{v}sin\theta$ is the azimuth reference signal.

### 2.3. GeoSta-GNSS-BFLSAR Resolution

In this section, the spatial resolution of GeoSta-GNSS-BFLSAR is briefly introduced on the basis of a Gaussian model in both the range direction and azimuth direction. The details are as follows:

The range and azimuth resolution can be given by Equations (8) and (9), respectively [29]:

(8) $$\sigma_{r}=\frac{\delta_{t}c}{2cos\frac{\beta }{2}}$$

(9) $$\sigma_{a}=\frac{\delta_{D}\lambda }{2\omega_{R}}$$

where $\delta_{t}$ and $\delta_{D}$ are the delay resolution and Doppler resolution, β is the bistatic angle, $\omega_{R}$ is the angular speed of the receiver with respect to the target point at slow time. The bistatic angle and slow time angular speed can be presented as:

(10) $$\beta =\frac{\sqrt{R_{r}{\left(\eta \right)}^{2}+R_{t}{}^{2}-B_{l}{\left(\eta \right)}^{2}}}{2R_{r}\left(\eta \right)R_{t}}$$

(11) $$\omega_{R}=\frac{\left|\left[I-\Phi_{R}\Phi_{R}{}^{T}\right]v\right|}{R_{r}\left(\eta \right)}$$

where $\;B_{l}\left(\eta \right)$ is baseline of the GeoSta-GNSS-BFLSAR system, I is the 3 × 3 unit matrix, $\Phi_{R}$ is the unit vector in the direction of the receiver’s line of sight for the target point at the slow time.

### 2.4. GeoSta-GNSS-BFLSAR Focusing Depth

In this section, the focusing depth of GeoSta-GNSS-BFLSAR imaging is derived. The details as follows:

In synthetic aperture’s edge, the maximum phase delay is given as:

(12) $$\Delta \phi =\frac{4\pi }{\lambda }\Delta R_{qmax}$$

The amount of range migration at aperture edge could be expressed as:

(13) $$\Delta R_{qmax}=\frac{\Delta R}{2cos\theta_{t}}\left(sec\frac{\theta_{SA}{}^{t}}{2}-1\right)+\frac{\Delta R}{2cos\theta_{r}}\left(sec\frac{\theta_{SA}{}^{r}}{2}-1\right)$$

where ΔR is the focusing depth, and $\theta_{SA}{}^{t}$ and $\theta_{SA}{}^{r}$ indicate the synthetic aperture angles of the transmitter and receiver, respectively.

As for the GeoSta-GNSS-BFLSAR system, the transmitter is located 36,000 Km above the Earth’s surface, $\theta_{t}≃0$ and $\theta_{SA}{}^{t}=0$. Therefore, the Equation (13) can be approximated as:

(14) $$\Delta R_{qmax}=\frac{\Delta R}{2cos\theta_{r}}\left(sec\frac{\theta_{SA}{}^{r}}{2}-1\right)$$

The variable $\sec\frac{\theta_{SA}{}^{r}}{2}$ could be calculated by employing the relationship between azimuth resolution and synthetic aperture angle, as listed below:

(15) $$\sigma_{a}=\frac{\lambda }{2sin\frac{\theta_{SA}{}^{r}}{2}cos\frac{\beta }{2}}$$

Therefore, by manipulating Equation (15), we got

(16) $$sin\frac{\theta_{SA}{}^{r}}{2}=\frac{1}{\sqrt{1-{\left(\frac{\lambda }{2\sigma_{a}cos\frac{\beta }{2}}\right)}^{2}}}$$

As such, by applying Equation (16), Equation (14) to Equation (12), the maximum phase delay could be rewritten as:

(17) $$\Delta \phi =\frac{4\pi }{\lambda }\Delta R_{qmax}\;=\frac{2\pi \Delta R}{\lambda cos\theta }\left(sec\frac{\theta_{SA}{}^{r}}{2}-1\right)\;=\frac{2\pi \Delta R}{\lambda cos\theta }\left[1/\sqrt{1-{\left(\frac{\lambda }{2\sigma_{a}cos\frac{\beta }{2}}\right)}^{2}}-1\right]$$

Due to the fact the maximum phase delay is no more than π, which can be formulated as:

(18) $$\Delta \phi =\frac{2\pi \Delta R}{\lambda cos\theta }\left[1/\sqrt{1-{\left(\frac{\lambda }{2\sigma_{a}cos\frac{\beta }{2}}\right)}^{2}}-1\right]\leq \pi$$

After manipulating Equation (18), the focusing depth ΔR can be given as:

(19) $$\Delta R\leq \frac{\lambda cos\theta }{2\left[1/\sqrt{1-{\left(\frac{\lambda }{2\sigma_{a}cos\frac{\beta }{2}}\right)}^{2}}-1\right]}$$

## 3. GeoSta-GNSS-BFLSAR Image Formation

Based on the signal model analysis mentioned above, a preliminary image formation algorithm dedicated for GeoSta-GNSS-BFLSAR is proposed in this section. There are two major challenges for GeoSta-GNSS-BFLSAR imaging. In GeoSta-GNSS-BFLSAR systems, due to the extremely high orbit of the transmitter, a long dwell time is needed to achieve sufficient image SNR, which may introduce large range cell migration. The second one is its serious spatial variance caused by specific geometry. 

**Figure 2:**
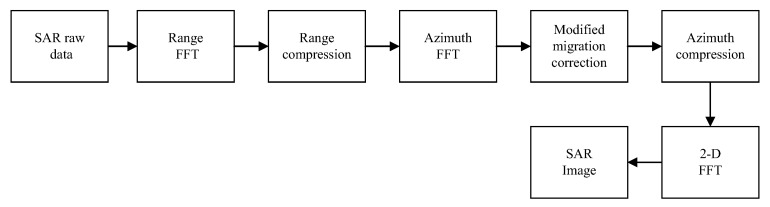
Block diagram of the proposed image formation algorithm for GeoSta-GNSS-BFLSAR.

The block diagram of the proposed image formation algorithm is shown in Figure 2. It mainly consists of three parts: range compression, modified migration correction and azimuth matched filtering. The details are as follows:

### 3.1. Range Compression

As the first step for GeoSta-GNSS-BFLSAR imaging processing, range compression is implemented to achieve the largest peak intensity in the range direction. Compared with conventional radar signals, GeoSta-GNSS-BFLSAR employs a BPSK-modulated pseudo-random sequence as the imaging signal. As such, a locally generated pseudo-random sequence with the same generator is applied as the base signal for range compression. It is noted that, however, the employment of a pseudo-random sequence has two sides. On one hand, compared with widely used linearly frequency modulated signal, pseudo-random sequence suffers from poor bandwidth, which is usually 5 MHz for single band GPS. One the other hand, the pseudo-random sequence benefits from good auto-correlation properties, in other words, extremely high peak power can be achieved via auto-correlation and inter-satellite interference can be eliminated via cross-correlation. The range compression reference signal is given by:

(20) $$h_{rcf}\left(t,\eta \right)=p\left(t\right)\cdot exp\left[-j2\pi f_{c}t\right]$$

The range compression is implemented in the azimuth time range frequency domain and the frequency domain output is represented as:

(21) $$S_{rc}\left(f_{t},\eta \right)=FFT_{t}\left[t,\eta \right]\cdot FFT_{t}{\left[h_{rcf}\left(t,\eta \right)\right]}^{*}\;=\sigma \cdot G\left(f_{t},\eta \right)\cdot exp\left[\frac{-j2\pi \left(f_{c}+f_{t}\right)R\left(\eta \right)}{c}\right]$$

where $G\left(f_{t},\eta \right)$ represents the spectrum of the cross-correlation of the received signal and the reference signal in the range direction.

### 3.2. Modified Migration Correction 

As for long dwell time forward looking observations, the range and azimuth cell migration are relatively high compared with the traditional scenario. An accurate and computationally efficient migration correction method is needed from both the performance side and efficiency perspectives. The conventional migration correction algorithms employ interpolation in every range direction for all the azimuth sample points, resulting in high accuracy and huge computational complexity at the same time. Aimed at reducing computational load, a frequency domain migration correction is implemented by applying a frequency domain-modified correction reference signal to the range compressed signal, i.e., Equation (21), after Fourier transformation in the azimuth direction, expressed as follows:

(22) $$\begin{array}{ll} S_{rc}\left(f_{t},f_{\eta }\right) & =\int S_{rc}\left(f_{t},\eta \right)\exp\left(-jf_{\eta }\eta \right)d\eta \\ & =\sigma \cdot G\left(f_{t},\eta \right)\cdot exp\left\{2\pi j\frac{f_{c}\left(R_{c}cos\theta +\left|T-P\right|\right)}{c}\right\}\\ & \cdot exp\left\{2\pi j\frac{cR_{C}cos\theta f_{\eta }^{2}}{2v^{2}\left(f_{c}+f_{t}\right)}-2\pi j\frac{f_{t}\left(R_{c}cos\theta +\left|T-P\right|\right)}{c}\right\}\\ & \cdot exp\left\{-2\pi jf_{\eta }\frac{R_{C}}{v}sin\theta \right\} \end{array}$$

The frequency domain modified correction factor is given by:

(23) $$\begin{array}{ll} H_{rcm}\left(f_{\tau },f_{\eta }\right) & =exp\left\{-2\pi j\frac{f_{c}\left(R_{ref}cos\theta \right)+\left|T-P\right|}{c}\right\}\\ & \cdot exp\left\{2\pi j\frac{cR_{c}cos\theta f_{\eta }^{2}}{2v^{2}\left(f_{c}+f_{t}\right)}-2\pi j\frac{f_{t}\left(R_{ref}cos\theta \right)+\left|T-P\right|}{c}\right\}\\ & \cdot exp\left\{-2\pi j\frac{f_{\eta }R_{ref}}{c}sin\theta \right\} \end{array}$$

where $R_{ref}$ is the slant range at the reference target point.

Applying convolution between Equations (22) and (23), the signal after modified correction processing will be presented as:

(24) $$\begin{array}{ll} S_{rcmc}\left(f_{t},f_{\eta }\right) & =FFT_{\eta }\left[S_{rc}\left(f_{t},f_{\eta }\right)\cdot H_{rcm}\left(f_{\tau },f_{\eta }\right)\right]\\ & =\sigma G\left(f_{t},f_{\eta }\right)\cdot exp\left\{-2\pi j\frac{cos\theta \left(R_{ref}-R_{c}\right)\left(f_{c},f_{t}\right)}{c}\right\}\\ & \cdot exp\left\{2\pi j\frac{ccos\theta f_{\eta }^{2}\left(R_{ref}-R_{c}\right)}{2v^{2}\left(f_{c}+f_{t}\right)}\right\}\cdot exp\left\{-2\pi j\frac{f_{\eta }sin\theta }{c}\left(R_{ref}-R_{c}\right)\right\} \end{array}$$

where $FFT_{\eta }\left[\cdot \right]$ indicates FFT in the azimuth direction.

It can be observed from Equation (24) that, once $R_{ref}$ equals $\;R_{c}$, all the phase terms will be removed. Therefore, in real imaging practice, for the sake of efficiency and accuracy, we make $R_{ref}=R_{c}$, and then, Equation (24) can be rewritten as:

(25) $$S_{rcmc}\left(f_{t},f_{\eta }\right)=\sigma G\left(f_{t},f_{\eta }\right)$$

### 3.3. Two Dimensional Inverse Fourier Transformation

Once the signal is corrected from both the range and azimuth perspective, an inverse Fourier transformation will be performed directly on Equation (25) in both the azimuth direction and range direction, leading to a focused image.

## 4. Simulation and Discussion

In this section, based on the analytical discussion mentioned above, a MATLAB-based simulation has been conducted for verification of the operational functionality and performance of the proposed imaging algorithm of the GeoSta-GNSS-BFLSAR system. The transmission signal with bandwidth of 5 MHz and platform of 36,000 Km is assumed in this simulation to imitate the real scenario. The integration time is assumed to be 1 second and receiver is mounted on a moving aircraft. The simulation parameters of GeoSta-GNSS-BFLSAR are listed in Table 1.

**Figure 3:**
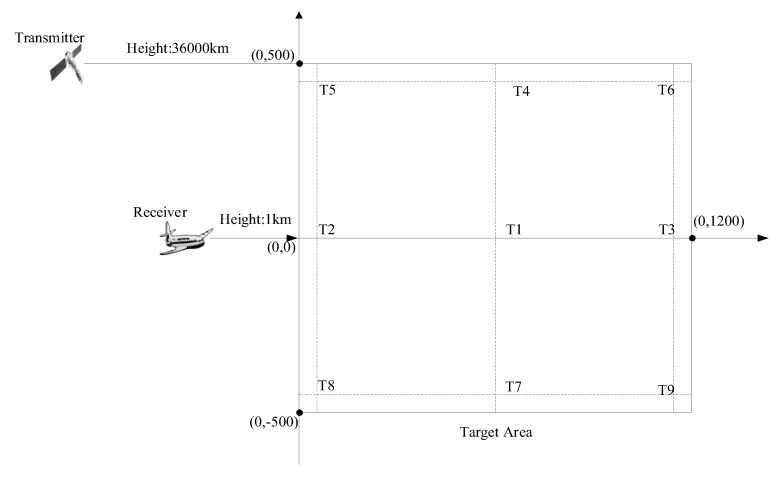
Topology of the nine point targets.

Nine point targets were simulated, separated in range by 500 m, and by 400 m in azimuth. The topology of the point targets is shown in Figure 3.

**Figure 4:**
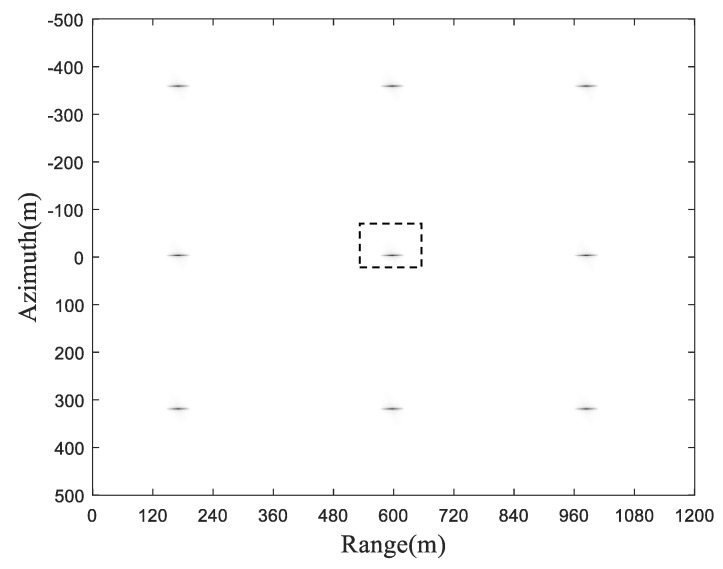
Final imaging result.

The final imaging result is shown in Figure 4.

**Figure 5:**
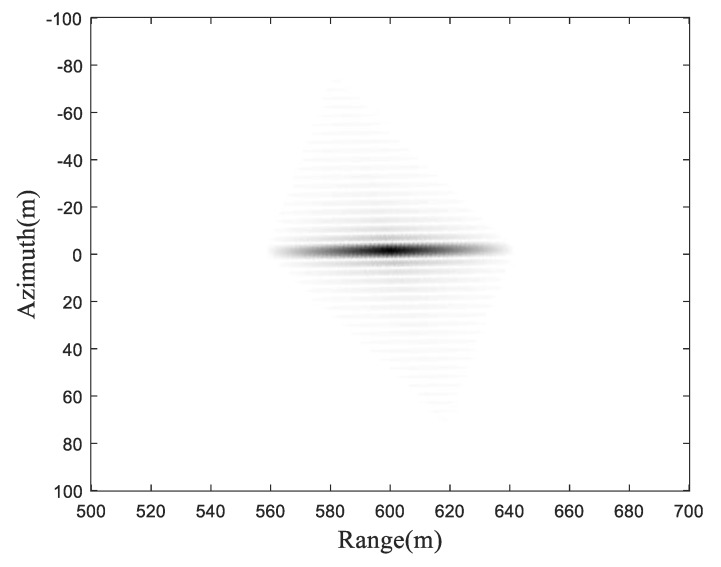
Zoomed image of the target point T1.

**Figure 6:**
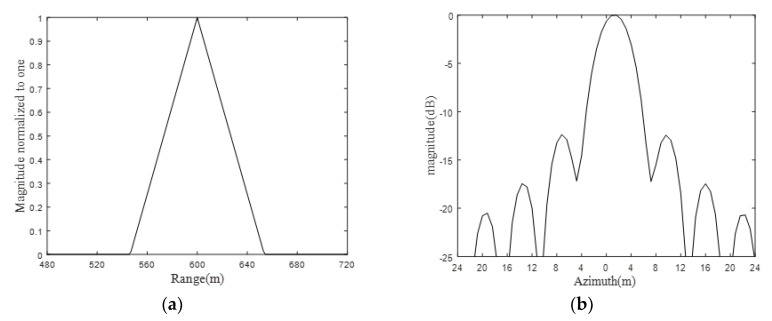
(**a**) Cross-section of the target point T1 in the range direction, (**b**) Cross-section of the target point T1 in the azimuth direction.

To further examine the focusing performance, for the sake of simplicity, we take the point at the center of the imaging scene, i.e., T1, for investigation (marked by the dashed rectangular box). The zoomed image of target point T1 is shown in Figure 5, while the cross-section in both the range direction and azimuth direction are shown in Figure 6.

It could be directly observed from Figure 6 that the cross section is rectangular and sine-shaped in the range direction and azimuth direction, respectively, which is in good accordance with the theoretical counterpart from a qualitative point of view. The quantitative analysis will be presented later.

In addition, for the sake of generality, the imaging parameters, e.g., PSLR and SLR of representative three target points, i.e., T1, T5 and T9 are listed in Table 2. 

It could be observed from Figure 6 and Table 2 that the image is fairly well focused at the point targets from both the scene center and scene edge perspectives.

**Figure 7:**
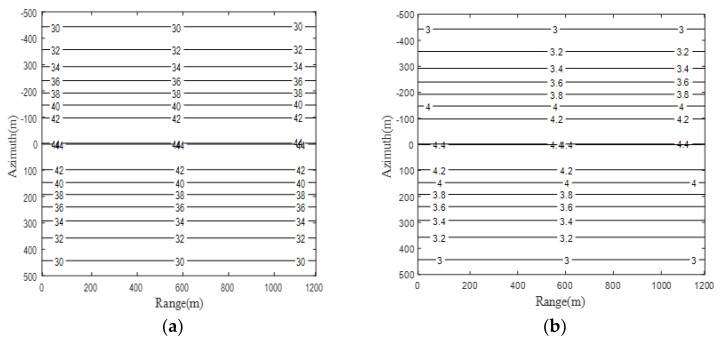
Theoretical spatial resolution of the proposed SAR configuration: (**a**) range resolution; (**b**) azimuth resolution.

Based on the mathematical analysis of resolution in Equations (8) and (9), simulation of resolution is implemented and the result is shown in Figure 7.

**Figure 8:**
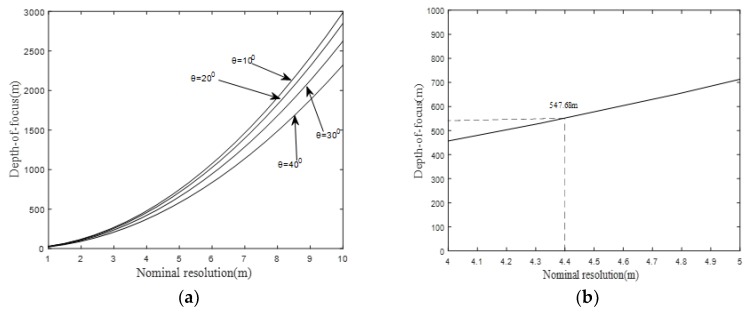
(**a**) Focusing depth values over the resolution with different forward looking angles; (**b**) zoom plot of Figure 8a at a forward-looking angle of 20 degrees around resolutions between 3 m to 5 m.

Bring on Figure 6 and Figure 7 together, it could be observed that the simulation result is in good agreement with theoretical analysis in terms of spatial resolution. With regards to focusing depth, according to Equation (19), a simulation has been conducted with ascending resolution and different forward-looking angles. The result is shown in Figure 8, where Figure 8a is the simulated curve of focusing depth with stepping resolutions and four typical forward-looking angles. Figure 8b is the zoom plot of Figure 8a at a forward-looking angle of 20 degrees around a resolution between 3 m to 5 m.

It can be observed from Figure 8a that, with ascending resolution, the focusing depth is also increasing in the case of a fixed forward-looking angle. Apart from that, in order to keep a fixed resolution, a smaller forward-looking angle is needed for better focusing depth.

**Figure 9:**
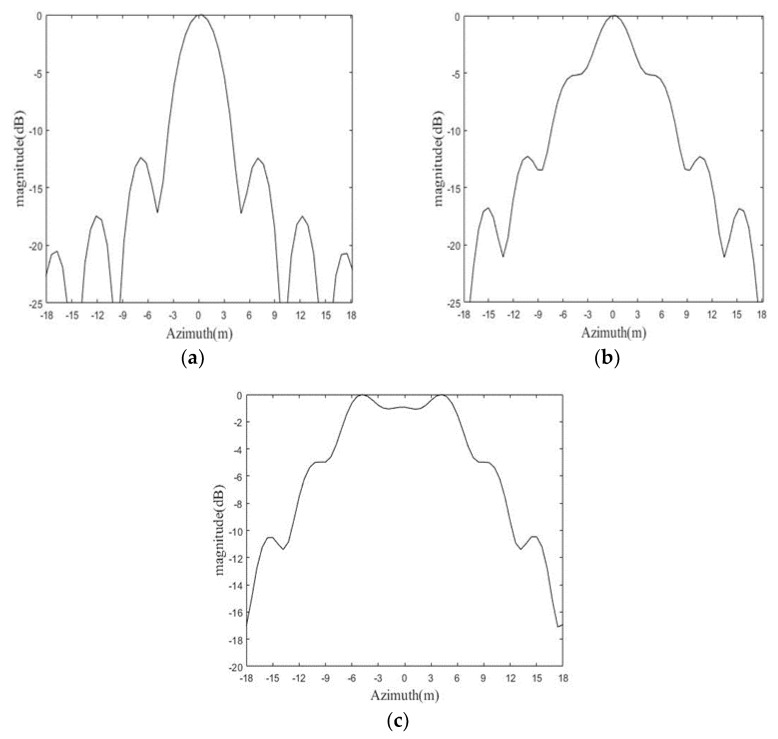
Cross-sections at azimuth direction of three targets at different range distances: (**a**) 0 m, (**b**) 510.68 m, (**c**) 1000 m.

In addition, at an arbitrary target point with azimuth resolution of 4.3 m, which is marked by the yellow dashed box in Figure 7a, the focusing depth of 510.68 can be easily obtained from Figure 8b, which indicates the focusing bound of our proposed imaging algorithm in the specific GeoSta-GNSS-BFLSAR configuration. To verify the correctness of analysis of focusing depth mentioned above, an extra simulation is performed with three target points at different slant range, listed as follows (Table 3):

The cross-sections of three simulated targets at azimuth direction are shown in Figure 9.

It can be found from Figure 9 that the targets are becoming defocused as the slant range grows. The target at reference slant range is well focused, while that beyond the focusing depth is seriously defocused, indicating the validity of the focusing depth analysis in this paper.

## 5. Conclusions

In this paper, a novel image formation algorithm has been presented for a newly proposed radar concept, i.e., GeoSta-GNSS-BFLSAR, where the transmitter is a geo-stationary navigation satellite and the receiver is mounted on a moving aircraft. 

To achieve the optimal efficiency, the proposed algorithm was implemented in frequency domain and constituted of three parts, including range compression, modified migration correction and 2-dimensional inverse Fourier transformation. Particularly, a modified migration correction factor has been specially designed to accommodate the long dwell time and spatial variance in GeoSta-GNSS-BFLSAR. The proposed algorithm has been analytically derived and proved fully functional by simulation. In addition, the radar performance and technical limitations of the proposed algorithm, i.e., resolution and focusing depth, were analytically demonstrated and numerically simulated. It could be found that the range resolution and azimuth resolution can be up to 44 m and 4.4 m, respectively, which has a high level of agreement with the imaging simulation results. The further indication of a focusing depth of 510.68 m allowed the conclusion that the limitations of this algorithm are with regards to the extent of the imaging scene that can be processed. 

To conclude, the above results prove the feasibility of the GeoSta-GNSS-BFLSAR system from a simulation level perspective and establish a foundation for the real application of such a radar scheme in the future. Our future work will focus on developing a large scale image formation algorithm.

## Figures and Tables

**Table 1:** Main simulation parameters of GeoSta-GNSSBFLSR system.

Parameter	Value
Transmitter Height	36,000 km
Receiver Height	2000 m
Receiver velocity	300 m/s
Receiver inclination Angle	20^o^
Carrier Frequency	1.602 GHz
Signal Bandwidth	5 MHz
PRF	1000 Hz

**Table 2:** Imaging parameters of three target points.

Target	Range		Azimuth	
	PSLR (dB)	ISLR (dB)	PSLR (dB)	ISLR (dB)
T1	−27.3	−19.4	−13.3	−10.2
T5	−27.2	−19.3	−13.2	−10.2
T9	−27.2	−19.3	−13.2	−10.2

**Table 3:** Three slant ranges of different targets.

Target	Slant Range (Relative to Reference Slant Range)
H1	0 m
H2	510.68 m
H3	1000 m
